# Supplementary material for: Splice-Junction-Based Mapping of Alternative Isoforms in the Human Proteome
Source: Cell Rep. Author manuscript; Available in PMC 2020 Jan 15. (PMC6961840; doi:10.1016/j.celrep.2019.11.026)
Supplement: 2 [file NIHMS1546469-supplement-2.zip › DF1/PXD006675/LeftCoronaryArtery/LeftCoronaryArtery_6_ELN_AQLLPGALAAAK.pdf]

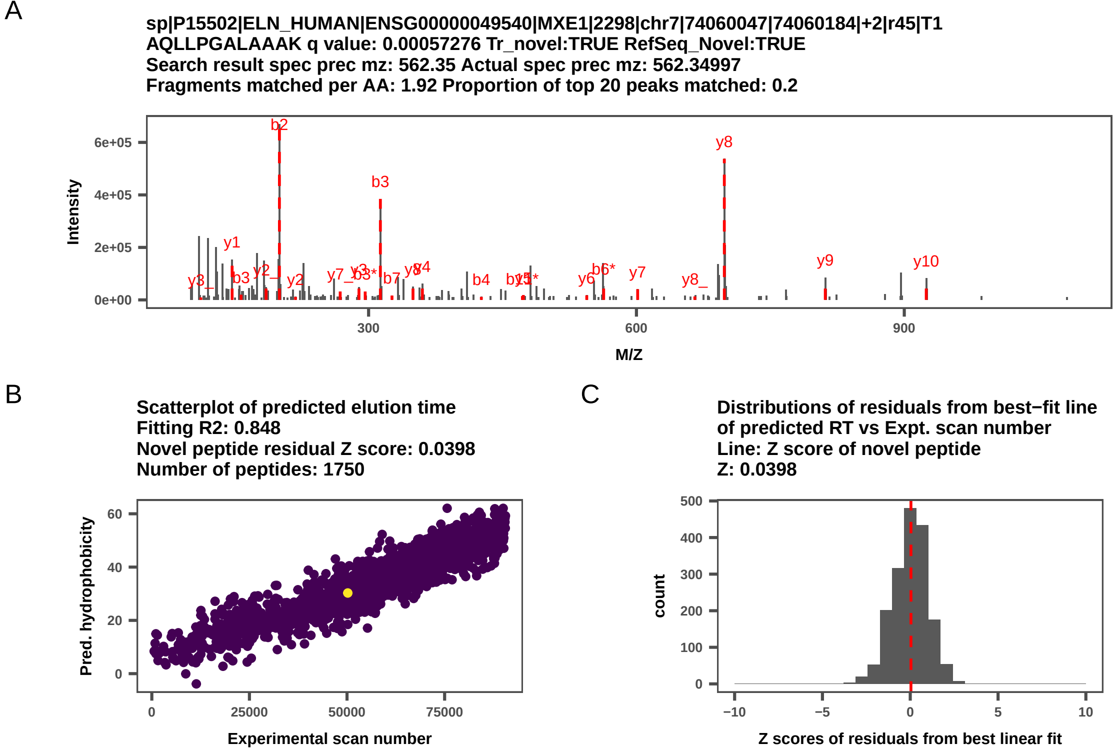
A
sp|P15502|ELN_HUMAN|ENSG00000049540|MXE1|2298|chr7|74060047|74060184|+2|r45|T1
AQLLPGALAAAK q value: 0.00057276 Tr_novel:TRUE RefSeq_Novel:TRUE
Search result spec prec mz: 562.35 Actual spec prec mz: 562.34997
Fragments matched per AA: 1.92 Proportion of top 20 peaks matched: 0.2
Intensity
6e+05
4e+05
2e+05
0e+00
b2
b3
y8
y1
y9
y10
b4
y6
b6*
y7
b7
y2
M/Z
300
600
900
B
Scatterplot of predicted elution time
Fitting R2: 0.848
Novel peptide residual Z score: 0.0398
Number of peptides: 1750
Pred. hydrophobicity
60
40
20
0
0
25000
50000
75000
Experimental scan number
C
Distributions of residuals from best−fit line
of predicted RT vs Expt. scan number
Line: Z score of novel peptide
Z: 0.0398
count
500
400
300
200
100
0
−10
−5
0
5
10
Z scores of residuals from best linear fit
